# Cytokine Profile in Children Following SARS-CoV-2 Infection: Preliminary Findings

**DOI:** 10.1097/INF.0000000000004558

**Published:** 2024-10-01

**Authors:** Danilo Buonsenso, Anna Camporesi, Gabriele Di Sante, Michela Sali, Maria del Carmen Pereyra Boza, Rosa Morello, Piero Valentini, Francesca Raffaelli, Lucie Rodriguez, Laura Gonzalez, Anette Johnsson, Constantin Habimana Mugabo, Tadepally Lakshmikanth, Petter Brodin

**Affiliations:** From the * Department of Woman and Child Health and Public Health, Fondazione Policlinico Universitario A. Gemelli IRCCS, Rome, Italy; † Centers for Global Health Research Studies, Università Cattolica del Sacro Cuore, Rome, Italy; ‡ Area Pediatrica, Dipartimento di Scienze della Vita e di Sanità Pubblica, Università Cattolica del Sacro Cuore, Rome, Italy; § Anesthesia and Intensive Care Unit, “Vittore Buzzi” Children’s Hospital, Milan, Italy; ¶ Department of Medicine and Surgery, Section of Human, Clinical and Forensic Anatomy, University of Perugia, Perugia, Italy; ∥ Dipartimento di Scienze di Laboratorio e Infettivologiche, Fondazione Policlinico Universitario “A. Gemelli,” IRCCS, Rome, Italy; ** Dipartimento di Scienze biotecnologiche di base, Cliniche intensivologiche e perioperatorie-Sezione di Microbiologia, Università Cattolica del Sacro Cuore, Rome, Italy; †† Dipartimento di Scienze Mediche e Chirurgiche, UOC di Malattie infettive, Fondazione Policlinico Universitario A. Gemelli IRCCS, Rome, Italy; ‡‡ Department of Women’s and Children’s Health, Karolinska Institutet, Solna, Sweden; §§ Medical Research Council Laboratory of Medical Sciences (LMS), Imperial College Hammersmith Campus, London, United Kingdom.

**Keywords:** long COVID, SARS-CoV-2, COVID-19, children, cytokine profile

## Abstract

We provide preliminary evidence that, also in children, Long coronavirus disease (COVID) may be characterized by a proinflammatory signature. Ten Long COVID patients, 7 convalescent subjects after COVID infection and 4 healthy controls were enrolled. When adjusted for sex, children with long COVID had statistically significant differences in the levels of Flt3L, CD5, uPA, CCL23, CD40 and TGFα. When adjusted for age, CCL23 levels remained statistically significant.

It is widely accepted, and demonstrated, that severe acute respiratory syndrome coronavirus 2 (SARS-CoV-2) infection can cause a long-lasting pattern of debilitating symptoms that are currently affecting millions of people worldwide.^1^ This condition, known as Long COVID, post-COVID condition or post-acute sequelae of SARS-CoV-2, is characterized by the persistence of signs and symptoms that were not present before SARS-CoV-2 infection and endure for at least 12 weeks, negatively impacting daily life.^2^

Long COVID has been extensively reported both in adults and children, particularly adolescents, and can follow even mild to asymptomatic infections or reinfections.^3^ Although the causes leading some patients to develop Long COVID are still unknown, a growing body of literature has documented that several biological abnormalities are evident in patients with Long COVID compared with healthy controls, including events associated with thromboinflammation, persistent immune activation and imbalance.^4–6^ However, these studies have been limited to adults only, and so far, no studies have attempted to provide a deep cytokine profile of children with a previous SARS-CoV-2 infection.

Therefore, in this study, we attempted to provide a preliminary assessment of cytokine profile in a cohort of children with previous SARS-CoV-2 infection, either fully recovered or experiencing Long COVID, with the aim of guiding future, larger research studies.

## METHODS

The study methodology is detailed in Supplemental Digital Content 1, http://links.lww.com/INF/F741.^7^

## RESULTS

Twenty-one patients were enrolled in the study: 10 for the Long COVID group, 7 convalescent subjects after COVID infection and 4 healthy controls. Supplemental Digital Content 2, http://links.lww.com/INF/F741, shows their baseline demographic characteristics, symptoms and antibody levels.

Cytokine levels among the different groups are shown in Supplemental Digital Content 2, http://links.lww.com/INF/F741. Interleukin (IL)2, IL22-RA1, IL20, IL33 had all results under limit of detection. IL 20RA, IL2-RB, IL1α, βNGF, IL24, IL13, IL4, LIF and NRTN had more than 80% of values under the limit of detection.

**FIGURE 1.**
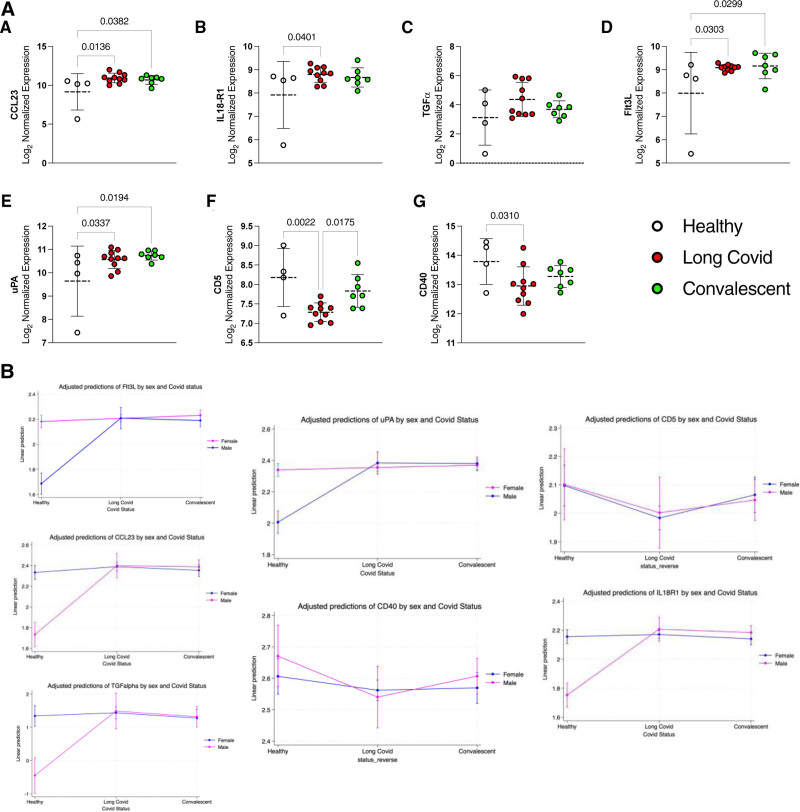
Cytokine expression by COVID‐19 status. A–G: Levels of different proteins in the 3 groups. A: Cytokine expression by COVID-19 status. B: Cytokine expression corrected by sex.

The univariate analysis of a group of cytokines, chemokines and receptors had a significant relationship with Long COVID status (see Supplemental Digital Content 3, http://links.lww.com/INF/F741 and Fig. 1A). Patients affected by Long COVID status show higher levels of Chemokine (C-C motif) ligand 23 (CCL23), transforming growth factor-alpha, IL 18 receptor 1 (IL18-R1) when compared with the other groups, while FMS‐like tyrosine kinase 3 ligand (FLT3L) correlated with the recovery from the symptoms. When adjusted for sex, Flt3L, CD5, uPA, CCL23, CD40 remained significant. Transforming growth factor-alpha, IL18R1 were close to significance, possibly due to the small sample size (Fig. 1B). When adjusted for age, CCL23 remained significant. Levels of the abovementioned cytokines differed also between sexes, mostly in the healthy patients but not in Long COVID (Supplemental Digital Content 4, http://links.lww.com/INF/F741).

None of them had correlation with temporal distance from and severity of primary infection (Supplemental Digital Content 5, http://links.lww.com/INF/F741). Among the subgroup of patients with Long COVID, we found that CD40 correlates with neurological symptoms, migraine, muscular symptoms, and CD5 correlates with post-exertional malaise (Supplemental Digital Content 6, http://links.lww.com/INF/F741).

## DISCUSSION

In this preliminary study, we attempted to perform a cytokine profiling of children after SARS-CoV-2 infection, including both patients with a clinical diagnosis of Long COVID and those fully recovered from the initial infection.

We discovered that CC chemokine ligand 23 [CCL23; also known as chemokine β8-1 (Ckβ8-1), myeloid progenitor inhibitory factor 1 and macrophage inflammatory protein 3 (MIP-3)] was significantly associated with Long COVID, even after adjusting for age and sex. CCL23 is a new member of the small CC chemokine family that has recently been associated with the pathophysiology of various inflammatory conditions.^8,9^ Interestingly, secreted CCL23 also functionally contributes to modulation of the immune response via promoting leucocyte trafficking as well as directing the migration of monocytes, macrophages and activated T lymphocytes to local sites of injury.^10^ Circulating CCL23 interacts with CC chemokine receptor 1 (CCR1), subsequent upregulating several adhesion molecules that promote the migration of circulating immune cells to the inflamed microenvironment.^11^ These findings suggest that, in line with adult Long COVID, there might be inflammatory microenvironments in pediatric Long COVID as well. In fact, CCL23 plays a pathological role in the development or progression of several inflammatory diseases, such as rheumatoid arthritis, chronic rhinosinusitis, chronic kidney dysfunction and systemic sclerosis.^12–16^ In this context, SARS-CoV-2 viral persistence is currently a leading hypothesis behind Long COVID. Considering that viral persistence has also been documented in children,^17^ the association of CCL23 with pediatric Long COVID suggests that similar events can happen also in children. Notably, CCL23 also boosts angiogenesis via promotion of endothelial cell migration through upregulation of several matrix metalloproteinases in the endothelium.^18,19^ Endotheliopathy and thromboinflammation have been strongly associated with adult Long COVID by several independent cohorts.^4^ More recently, circulating CCL23 levels have also been found to be possible biomarker to diagnose acute inflammatory reactions in the brain after cerebral damage,^20^ an interesting data considering that patients with Long COVID also develop neurocognitive symptoms and brain involvement has been demonstrated in adults and children following SARS-CoV-2 infection. To further reinforce our hypothesis that CCL23 may be a marker of pediatric Long COVID, CCL23 is less expressed with age,^21^ while in our series, it is higher in patients than controls, despite being older, suggesting therefore a true and strong correlation of this marker with Long COVID and, in general, SARS-CoV-2 infection. In addition, the same findings have been found in adults with post-traumatic stress disorder, a condition that shares several clinical similarities with Long COVID.^22^ In Supplemental Digital Content 7, http://links.lww.com/INF/F741, we have provided further discussion about the other cytokines that were differently expressed by the cohorts, but not after adjustment of sex and age.

These findings may also be interpreted considering the observed outbreaks of severe infections after the pandemic, including invasive group A Streptococcus infection, brain abscesses and acute hepatitis.^23^ So far, most of these events have been linked with a so-called immunity debt, meaning a decline of immunity to certain pathogens due to lack of exposure to them during the periods of social restrictions. However, there is evidence of reduced anti-RSV IgG antibodies only in pregnant women and their newborns, but no other evidence has been documented so far in support of the immunity debt.^24^ On the other hand, adult studies have clearly documented post-COVID immune activation, which apparently resolves within 2 years.^25^ In this regard, our data, along with a previous publication^26^ suggest that immune phenomena may also happen in children and adolescents following SARS-CoV-2 infection, and so far, it remains unknown how this possibly transient immune activation can be associated with more severe outcomes when a new infection is encountered in this specific “immunological window.” Although this is only a hypothesis, we do believe that these phenomena are worth further investigations.

The main limitation of this study is represented by the low number of patients included, particularly among the healthy control group, which moreover was significantly younger compared with the others. This was due to the difficulty in accessing healthcare settings during the early periods of the pandemic, the only reasonable period where children may have been realistically naive from SARS-CoV-2 infection. Nevertheless, we have compensated the different age group of healthy controls by correcting for age in the multivariate analyses. In addition, for some of the cytokines showing statistically significant differences in Long COVID versus controls, there is no expected age-dependent expression.

In conclusion, our preliminary findings suggest that children with previous SARS-CoV-2 infection, and particularly those with Long COVID, exhibit altered cytokine expression patterns involved in T cell homeostasis and coagulation, in line with adult studies. These results underscore the importance of immunologic investigations in pediatric cohorts and support for larger-scale studies employing comprehensive immunological approaches, including proteomic and epigenetic approaches.

## Supplementary Material


