# Supplementary material for: Cytokine Profile in Children Following SARS-CoV-2 Infection: Preliminary Findings
Source: Pediatr Infect Dis J. 2024 Oct 1;44(1):54–7. doi: 10.1097/INF.0000000000004558 (PMC11627325; doi:10.1097/INF.0000000000004558)
Supplement: Supplementary file 1 [file inf-44-054-s001.pdf]

Supplementary materials

Supplemental Digital Content 1

Study population and setting

This study is preliminary immunological sub-analysis of a prospective follow-up study of children with microbiologically confirmed SARS-CoV-2 infection evaluated in person at a referral pediatric post-covid clinic in Rome, Italy. As previously published, the study population is a cohort of pediatric patients younger than 19 years old with laboratory Sars-Cov-2 infection (between 01/02/2020 to 30/04/2021), referred to our public post-Covid outpatient unit from our Emergency Department, admission ward or family pediatricians in the region/in Rome (3).

Children with a history of SARS-CoV-2 infection were assessed in our clinic at least 8 weeks following the initial infection and were classified as either "fully recovered" from the infection (reporting no persisting signs or symptoms and full return to pre-covid activity levels) or as "Long Covid". Long Covid was defined as the persistence of symptoms for at least eight weeks after initial infection, which had a negative impact on daily life, and other possible diagnoses excluded according to the definition provided by the WHO (2). All patients with Long Covid underwent additional tests to exclude other diagnoses that might have explained their persisting symptoms (eg. anemia, autoimmune diseases, parasitic disorders, hypo or hyperthyroidism, etc).

For each patient we collected information on demographics (age, gender, and pre-existing conditions severity of acute infection (as previously defined (3), main persisting symptoms, including fatigue, cardiovascular (e.g. tachycardia and palpitations), respiratory (dyspnoea or chronic cough), post exertional malaise (PEM), headache, neurocognitive symptoms, musculoskeletal problems and skin rashes. Data on the dominant circulating variant at the time of infection was collected from the report coordinated by the Italian National Institute of Health (7).

Blood samples were taken from cases during an outpatient visit and (within 30 minutes from the collection) were centrifuged at 3200 rpm for 15 minutes; successively, plasma samples were immediately stored at -80 °C.

Plasma protein profiling by Olink

Plasma protein data was generated using by Olink assay, a proximity extension assay (Olink AB, Uppsala) (Lundberg, M et al. Homogeneous antibody-based proximity extension assays provide sensitive and specific detection of low-abundant proteins in human blood. *Nucleic Acids Res* **39**, e102–e102 (2011). 20uL of plasma from each sample was thawed and analyzed using a Target™ Inflammation panel (Olink AB), at the Affinity Proteomics-Stockholm unit, Science for Life Laboratory, Stockholm, Sweden. In these assays, plasma proteins are dually recognized by pairs of antibodies coupled to a cDNA-strand that ligates when brought into proximity by its target, extended by a polymerase and detected using a Biomark HD 96.96 dynamic PCR array (Standard BioTools Inc.). The data was analysed using Olink NPX Manager 3.1.0.393.

Statistical analyses

Categorical variables were described as frequencies and percentages, and continuous variables were expressed as median (interquartile range). To investigate the relationship between cytokine levels and COVID status (Long COVID, Convalescent, Healthy without prior COVID contact), we log-transformed cytokine values and utilized linear regression for complete data above the LOD (Limit of Detection) and Tobit regression for left-censored data below the LOD. Each cytokine was initially examined in a univariate model, followed by multivariate models that included age and sex as covariates. Results from models with significant covariate effects are presented. Two tailed tests were used and significance was set at 0.05. Data were analysed with Stata BE v. 18 (StataCorp. 2023. Stata Statistical Software: Release 18. College Station, TX: StataCorp LLC).

Ethical approval

The study is part of larger, prospective, multisectorial follow-up study of children with SARS-CoV-2 infection, approved by the local ethics committee (Ethic approval ID4518, Prot0040139/21), and informed consent was provided. Written and informed consent was obtained from parents/caregivers and from children older than 5 years of age, according to local guidance of the ethic committees.

Supplemental Digital Content 2: baseline demographic characteristics, symptoms and antibody levels of the cohort. Data are expressed as frequencies and percentages, or median (interquartile range). CV: cardiovascular. P.E.M.: Post-Exertional Malaise. EBV: Epstein-Barr Virus.

	Total	Long Covid	Healthy	Convalescent	p-value
	N=21	N=10	N=4	N=7	
Male sex	5 (23.8%)	1 (10.0%)	1 (25.0%)	3 (42.9%)	0.29
Age, years	11.0 (3.0-13.0)	13.0 (11.0-14.0)	3.0 (2.0-3.0)	8.0 (2.0-11.0)	0.005
Symptoms					
Fatigue	4 (19.0%)	4 (40.0%)	0 (0.0%)	0 (0.0%)	0.066
CV symptoms	4 (19.0%)	4 (40.0%)	0 (0.0%)	0 (0.0%)	0.066
Respiratory symptoms	2 (9.5%)	2 (20.0%)	0 (0.0%)	0 (0.0%)	0.3
PEM	6 (28.6%)	6 (60.0%)	0 (0.0%)	0 (0.0%)	0.01
Neurologic symptoms	4 (19.0%)	4 (40.0%)	0 (0.0%)	0 (0.0%)	0.066
Sleep disturbances	4 (19.0%)	4 (40.0%)	0 (0.0%)	0 (0.0%)	0.066
Muscular symptoms	4 (19.0%)	4 (40.0%)	0 (0.0%)	0 (0.0%)	0.066
Cephalalgia	5 (23.8%)	5 (50.0%)	0 (0.0%)	0 (0.0%)	0.027
Nucleosome	311.8 (72.3-489.6)	264.3 (72.3-487.1)	283.5 (181.8-504.0)	311.8 (25.3-544.6)	0.86
IgG_bau	146.5 (65.1-263.4)	146.5 (55.0-215.8)	0.0 (0.0-133.9)	251.7 (87.6-335.6)	0.057
IgG_au	36.3 (16.2-65.3)	36.3 (13.6-53.5)	0.0 (0.0-33.2)	62.4 (21.7-83.2)	0.057
IgM	0.3 (0.0-2.4)	0.8 (0.0-2.7)	0.0 (0.0-0.0)	0.7 (0.0-3.0)	0.22
IgA	0.0 (0.0-0.0)	0.0 (0.0-0.0)	0.0 (0.0-0.0)	0.0 (0.0-0.0)	
EBV_IgG	296.1 (203.8-503.5)	385.8 (224.6-674.8)	389.2 (290.5-999.3)	166.4 (118.8-312.8)	0.11
EBV_ IgM	1323.8 (866.0-1932.0)	1273.1 (942.8-2116.9)	1172.0 (605.9-1881.1)	1526.1 (762.8-1983.0)	0.81
anti-N_IgG	13.1 (4.7-23.3)	18.2 (6.5-23.1)	1.8 (1.5-9.0)	22.1 (5.0-33.3)	0.14
Distance from primary infection, days	141.0 (83.0-179.0)	149.0 (93.0-179.0)	-	99.0 (67.0-167.0)	0.47
Severity of the primary infection			-		0.001
Asymptomatic	3 (14.3%)	0 (0.0%)		3 (42.9%)	
Mild	14 (66.7%)	9 (90.0%)		4 (57.1%)	
Severe	1 (4.8%)	1 (10.0%)		0 (0.0%)	
Missing	3 (14.3%)	0 (0.0%)		0 (0.0%)	

Supplemental Digital Content 2: cytokine levels among groups.

Colonna1	Total	Healthy	Long Covid	Convalescent
	N=21	N=4	N=10	N=7
IL8	6.4 (5.1-7.8)	6.0 (4.3-6.8)	6.7 (5.1-11.1)	6.5 (5.1-7.8)
VEGFA	11.9 (11.5-12.2)	12.4 (10.2-13.3)	11.8 (11.5-12.0)	12.0 (11.4-12.8)
CD8A	9.6 (8.4-10.1)	9.1 (7.9-10.1)	9.7 (9.3-10.2)	9.3 (7.7-10.0)
MCP-3	2.7 (2.1-3.3)	3.0 (2.5-3.4)	2.8 (2.1-6.6)	2.3 (2.1-3.3)
GDNF	2.5 (2.5-2.5)	2.5 (2.5-2.5)	2.5 (2.5-2.7)	2.5 (2.5-2.5)
CDCP1	2.5 (2.3-2.6)	2.5 (1.5-2.9)	2.4 (2.2-2.5)	2.6 (2.4-2.7)
log_CDCP1	0.9 (0.8-1.0)	0.9 (0.3-1.0)	0.9 (0.8-0.9)	1.0 (0.9-1.0)
CD244	7.8 (7.1-8.3)	8.4 (7.3-9.0)	7.2 (6.9-8.1)	8.1 (7.5-8.3)
IL7	4.8 (3.8-5.5)	4.9 (3.3-5.9)	4.4 (3.6-5.0)	4.8 (4.2-5.5)
OPG	10.0 (9.7-10.3)	9.9 (8.1-10.1)	10.0 (9.7-10.3)	10.2 (9.7-10.4)
LAP TGF-beta	9.5 (9.2-10.2)	9.7 (8.8-10.4)	9.3 (9.1-10.2)	9.8 (9.3-10.3)
uPA	10.7 (10.4-10.8)	10.2 (8.7-10.6)	10.6 (10.4-10.8)	10.8 (10.7-11.0)
IL6	3.3 (2.6-3.6)	3.0 (2.6-3.5)	3.5 (2.6-4.1)	3.2 (3.0-3.5)
IL-17C	2.8 (2.7-3.5)	3.9 (2.9-4.3)	2.8 (2.8-3.5)	2.7 (2.6-3.3)
MCP-1	12.3 (11.4-12.9)	12.3 (9.9-12.6)	12.3 (11.4-13.5)	12.3 (11.4-12.8)
IL-17A	3.4 (3.2-3.6)	3.7 (3.4-4.4)	3.3 (3.2-3.5)	3.3 (3.2-3.6)
CXCL11	8.9 (8.4-10.0)	9.3 (8.1-10.1)	8.5 (8.0-9.6)	9.7 (8.7-10.5)
AXIN1	6.6 (4.3-8.7)	7.5 (5.0-8.6)	5.6 (4.0-9.3)	8.2 (6.2-9.2)
TRAIL	7.5 (7.3-7.8)	7.8 (6.1-8.2)	7.5 (7.3-7.8)	7.5 (7.2-7.6)

IL-20RA	2.6 (2.6-2.6)	2.6 (2.6-2.6)	2.6 (2.6-2.6)	2.6 (2.6-2.6)
CXCL9	6.7 (6.6-7.2)	6.8 (5.3-8.4)	6.6 (6.2-7.0)	7.0 (6.6-7.3)
CST5	6.2 (5.7-6.3)	5.8 (4.6-6.1)	6.1 (5.7-6.4)	6.3 (6.0-6.4)
IL-2RB	3.0 (3.0-3.0)	3.0 (3.0-3.0)	3.0 (3.0-3.0)	3.0 (3.0-3.0)
IL-1 alpha	-0.4 (-0.4--0.4)	-0.4 (-0.4--0.4)	-0.4 (-0.4--0.3)	-0.4 (-0.4--0.4)
OSM	6.7 (5.4-7.7)	6.1 (3.7-7.3)	7.0 (5.9-8.7)	6.5 (5.2-7.4)
IL2	2.4 (2.4-2.4)	2.4 (2.4-2.4)	2.4 (2.4-2.4)	2.4 (2.4-2.4)
CXCL1	11.8 (11.4-12.1)	11.9 (11.3-12.5)	11.8 (11.3-12.0)	11.7 (11.4-12.1)
TSLP	2.7 (2.7-2.7)	2.7 (2.7-2.8)	2.7 (2.7-2.7)	2.7 (2.7-2.7)
CCL4	7.6 (7.1-7.9)	7.3 (6.1-7.9)	7.7 (7.4-8.2)	7.7 (7.1-7.9)
CD6	7.2 (6.9-7.7)	7.4 (6.3-8.0)	7.1 (6.7-7.3)	7.1 (7.0-8.0)
SCF	9.9 (9.6-9.9)	9.6 (7.5-9.9)	9.9 (9.6-10.0)	9.8 (9.6-10.0)
IL18	9.4 (9.1-10.3)	9.3 (7.9-9.9)	9.2 (8.8-10.2)	10.3 (9.1-11.4)
SLAMF1	3.0 (3.0-3.1)	3.0 (3.0-3.0)	3.0 (3.0-3.1)	3.0 (3.0-3.3)
TGF-alpha	3.8 (3.3-4.7)	3.4 (1.7-4.5)	4.0 (3.4-5.8)	3.8 (3.2-4.0)
MCP-4	15.0 (14.5-15.6)	15.3 (13.1-15.6)	15.0 (14.3-15.6)	14.8 (14.5-15.5)
CCL11	8.2 (7.7-8.6)	8.5 (6.7-8.7)	8.1 (7.7-8.5)	8.0 (7.7-8.9)
TNFSF14	8.1 (7.3-8.8)	8.0 (6.2-8.4)	8.9 (7.3-9.0)	7.8 (6.9-8.4)
FGF-23	1.0 (0.6-1.1)	1.1 (0.6-1.7)	0.8 (0.6-1.1)	1.0 (0.5-1.0)
IL-10RA	1.9 (1.7-2.2)	1.7 (1.5-1.9)	1.9 (1.8-2.2)	2.0 (1.6-2.2)
FGF-5	1.5 (1.4-1.6)	1.4 (1.2-1.6)	1.5 (1.4-1.7)	1.5 (1.2-1.6)
MMP-1	14.9 (14.3-15.2)	14.3 (11.9-15.2)	15.0 (14.3-15.7)	14.7 (13.4-15.0)
LIF-R	4.1 (3.9-4.3)	4.0 (2.7-4.2)	4.2 (3.7-4.4)	4.1 (4.0-4.2)

FGF-21	3.5 (2.9-4.3)	3.8 (2.7-4.2)	3.8 (3.1-4.3)	3.3 (2.4-4.3)
CCL19	9.5 (8.8-9.9)	9.9 (7.8-10.7)	9.0 (8.4-9.9)	9.5 (9.1-9.8)
IL-15RA	2.2 (2.1-2.4)	2.2 (1.8-2.4)	2.2 (2.1-2.4)	2.3 (2.0-2.5)
IL-10RB	7.5 (7.4-7.8)	7.5 (5.6-7.8)	7.5 (7.4-7.7)	7.7 (7.5-8.1)
IL-22 RA1	4.5 (4.5-4.5)	4.5 (4.5-4.5)	4.5 (4.5-4.5)	4.5 (4.5-4.5)
IL-18R1	8.7 (8.5-8.9)	8.6 (7.2-8.7)	8.9 (8.5-9.1)	8.6 (8.4-8.9)
PD-L1	6.8 (6.4-7.3)	7.1 (6.7-7.7)	6.6 (6.2-7.3)	7.2 (6.3-7.3)
Beta-NGF	0.1 (0.1-0.1)	0.2 (0.1-0.3)	0.1 (0.1-0.1)	0.1 (0.1-0.1)
CXCL5	13.3 (12.6-13.5)	13.4 (13.0-13.6)	12.6 (12.2-13.3)	13.5 (12.9-13.5)
TRANCE	6.2 (5.5-6.5)	6.5 (4.8-6.7)	5.8 (5.3-6.2)	6.4 (5.6-6.6)
HGF	10.1 (9.8-10.6)	10.3 (8.1-10.6)	10.2 (9.8-10.7)	10.1 (9.7-10.5)
IL-12B	8.8 (8.3-8.9)	8.7 (7.1-9.0)	8.7 (7.9-8.9)	8.8 (8.4-9.3)
IL-24	3.0 (3.0-3.0)	3.0 (3.0-3.0)	3.0 (3.0-3.0)	3.0 (3.0-3.0)
IL13	2.4 (2.4-2.4)	2.4 (2.4-2.4)	2.4 (2.4-2.4)	2.4 (2.4-2.4)
ARTN	2.0 (2.0-2.0)	2.0 (2.0-2.1)	2.0 (2.0-2.1)	2.0 (2.0-2.0)
MMP-10	9.1 (8.4-9.8)	9.6 (7.4-10.0)	8.9 (8.4-9.6)	9.4 (7.6-10.3)
IL10	3.7 (3.3-3.8)	4.1 (3.1-4.5)	3.6 (3.3-3.7)	3.6 (2.7-3.8)
TNF	4.4 (3.9-4.7)	4.7 (3.7-5.3)	4.0 (3.9-4.7)	4.5 (4.1-4.6)
CCL23	10.8 (10.2-11.0)	10.2 (7.9-10.4)	10.9 (10.6-11.3)	10.9 (10.3-11.0)
CD5	7.4 (7.2-7.9)	8.3 (7.7-8.7)	7.3 (7.0-7.4)	7.7 (7.4-8.2)
CCL3	7.1 (6.7-8.0)	6.9 (5.7-7.8)	7.4 (6.4-8.7)	7.2 (6.9-8.0)
Flt3L	9.1 (8.9-9.2)	8.7 (7.0-9.0)	9.1 (9.0-9.2)	9.2 (8.9-9.6)
CXCL6	11.4 (10.6-12.0)	11.6 (10.1-12.5)	10.7 (10.4-12.0)	11.7 (11.4-12.0)

CXCL10	9.6 (9.2-10.4)	10.1 (8.0-11.9)	9.5 (8.8-10.0)	9.6 (9.3-11.3)
4E-BP1	11.3 (7.5-11.6)	10.5 (7.8-11.8)	11.0 (7.1-11.3)	11.4 (11.2-11.7)
IL-20	1.3 (1.3-1.3)	1.3 (1.3-1.3)	1.3 (1.3-1.3)	1.3 (1.3-1.3)
SIRT2	8.4 (5.2-9.7)	8.3 (5.8-9.9)	7.1 (5.1-9.9)	8.4 (5.2-9.7)
CCL28	2.7 (2.6-3.6)	3.6 (2.7-4.6)	2.7 (2.5-3.4)	2.7 (2.6-3.6)
DNER	9.6 (9.4-9.7)	9.7 (8.0-10.0)	9.4 (9.3-9.7)	9.7 (9.6-9.8)
EN-RAGE	5.4 (5.2-6.9)	5.3 (3.8-5.7)	6.2 (5.3-7.6)	5.3 (5.1-5.6)
CD40	13.1 (12.7-13.7)	14.0 (13.2-14.4)	12.9 (12.5-13.1)	13.4 (12.9-13.5)
IL33	2.1 (2.1-2.1)	2.1 (2.1-2.1)	2.1 (2.1-2.1)	2.1 (2.1-2.1)
IFN-gamma	7.2 (6.7-8.1)	7.8 (6.2-9.2)	7.2 (6.7-7.4)	7.3 (6.6-8.3)
FGF-19	8.7 (7.6-9.0)	7.8 (5.9-9.0)	8.8 (7.9-9.0)	8.7 (7.4-9.1)
IL4	2.6 (2.6-2.6)	2.6 (2.6-2.6)	2.6 (2.6-2.6)	2.6 (2.6-2.6)
LIF	1.0 (1.0-1.0)	1.0 (1.0-1.3)	1.0 (1.0-1.0)	1.0 (1.0-1.0)
NRTN	1.9 (1.9-1.9)	1.9 (1.9-1.9)	1.9 (1.9-1.9)	1.9 (1.9-1.9)
MCP-2	10.2 (9.7-10.5)	10.7 (8.9-11.0)	10.0 (9.7-10.5)	10.1 (9.5-10.5)
CASP-8	6.8 (6.1-7.5)	6.8 (5.2-7.8)	6.9 (6.5-7.9)	6.3 (5.4-7.4)
CCL25	6.4 (6.1-6.5)	6.5 (5.3-6.7)	6.3 (5.7-6.4)	6.4 (6.3-6.5)
CX3CL1	4.8 (4.4-5.1)	4.8 (3.1-5.0)	4.9 (4.5-5.1)	4.4 (4.3-5.4)
TNFRSF9	7.3 (7.2-7.9)	7.6 (5.9-8.2)	7.2 (6.5-7.6)	7.6 (7.2-8.2)
NT-3	3.5 (3.3-3.8)	3.6 (2.8-3.8)	3.5 (3.3-3.8)	3.5 (3.2-3.9)
TWEAK	9.5 (9.2-9.8)	9.5 (7.4-9.8)	9.4 (9.2-9.6)	9.8 (9.4-9.9)
CCL20	8.1 (7.3-8.9)	8.4 (7.1-9.7)	8.0 (7.2-8.3)	8.5 (8.1-8.9)
ST1A1	5.2 (4.2-5.7)	5.6 (4.7-5.7)	4.9 (3.8-5.9)	5.2 (4.6-5.8)

STAMBP	7.8 (5.7-9.9)	8.2 (6.0-9.8)	6.9 (5.6-10.0)	8.2 (6.0-9.9)
IL5	2.7 (2.7-2.8)	2.7 (2.7-2.8)	2.7 (2.7-5.6)	2.7 (2.7-2.7)
ADA	7.8 (7.0-8.7)	7.7 (6.4-9.4)	7.4 (6.4-7.9)	8.7 (7.5-8.8)
TNFB	5.7 (5.4-5.9)	5.9 (4.4-6.3)	5.5 (4.9-5.8)	5.7 (5.6-6.3)
CSF-1	10.3 (10.2-10.4)	10.4 (8.3-10.5)	10.3 (10.2-10.4)	10.3 (10.2-10.4)

Supplemental Digital Content 3: Univariate analysis results for cytokine levels and Long Covid status.

Citokine	Status	Coefficient	P value	95% Conf.	Interval
**CCL23**	Long Covid	0.206	0.017	0.041	0.371
	Convalescent	0.184	0.04	0.009	0.360
**IL18R1**	Long Covid	0.119	0.04	0.006	0.232
	Convalescent	0.104	0.084	-0.015	0.223
**TGFalpha**	Long Covid	0.546	0.047	0.009	1.084
	Convalescent	0.399	0.158	-0.170	0.969
**Flt3L**	Long Covid	0.149	0.031	0.015	0.284
	Convalescent	0.155	0.034	0.013	0.298
**uPA**	Long Covid	0.101	0.034	0.008	0.194
	Convalescent	0.117	0.021	0.019	0.216
**CD40**	Long Covid	-0.062	0.033	-0.119	-0.005
	Convalescent	-0.036	0.216	-0.096	0.023
**CD5**	Long Covid	-0.113	0.002	-0.180	-0.045
	Convalescent	-0.041	0.237	-0.112	0.029

Supplemental Digital Content4: Contrasts between sexes at different levels of Covid Status for selected cytokines

Flt3L	Contrast	Std. err.	[95% conf.	interval]	p value
male@status_reverse					
(Male vs Female) Healthy	-0.495	0.046	-0.593	-0.397	<0.001
(Male vs Female) Long Covid	0.001	0.042	-0.089	0.091	0.970
(Male vs Female) Convalescent	-0.042	0.031	-0.107	0.023	0.190
**CD5**	**Contrast**	**Std. err.**	**[95% conf.**	**interval]**	**p value**
male@status_reverse					
(Male vs Female) Healthy	0.004	0.068	-0.140	0.148	0.95
(Male vs Female) Long Covid	0.018	0.062	-0.113	0.150	0.77
(Male vs Female) Convalescent	-0.019	0.045	-0.114	0.077	0.68
**uPA**	**Contrast**	**Std. err.**	**[95% conf.**	**interval]**	**p value**

male@status_reverse					
(Male vs Female) Healthy	-0.333	0.038	-0.414	-0.251	<0.001
(Male vs Female) Long Covid	0.029	0.035	-0.046	0.104	0.41
(Male vs Female) Convalescent	0.011	0.025	-0.043	0.065	0.67
**CCL23**	**Contrast**	**Std. err.**	**[95% conf.**	**interval]**	**p value**
male@status_reverse					
(Male vs Female) Healthy	-0.601	0.064	-0.736	-0.465	<0.001
(Male vs Female) Long Covid	0.010	0.058	-0.114	0.134	0.86
(Male vs Female) Convalescent	0.034	0.042	-0.056	0.124	0.43
**CD40**	**Contrast**	**Std. err.**	**[95% conf.**	**interval]**	**p value**
male@status_reverse					
(Male vs Female) Healthy	0.064	0.053	-0.049	0.178	0.24
(Male vs Female) Long Covid	-0.022	0.048	-0.125	0.081	0.65
(Male vs Female) Convalescent	0.038	0.035	-0.037	0.112	0.3
**TGFalpha**	**Contrast**	**Std. err.**	**[95% conf.**	**interval]**	**p value**
male@status_reverse					
(Male vs Female) Healthy	-1.791	0.291	-2.412	-1.171	<0.001
(Male vs Female) Long Covid	0.051	0.266	-0.516	0.618	0.85
(Male vs Female) Convalescent	0.0418	0.193	-0.369	0.452	0.83
**IL18R1**	**Contrast**	**Std. err.**	**[95% conf.**	**interval]**	**p value**
male@status_reverse					
(Male vs Female) Healthy	-0.403	0.044	-0.498	-0.308	<0.001
(Male vs Female) Long Covid	0.036	0.040	-0.050	0.123	0.38
(Male vs Female) Convalescent	0.044	0.029	-0.019	0.106	0.15

Supplemental Digital Content 5a and 5b. Cytokine profile by time since infection and covid status

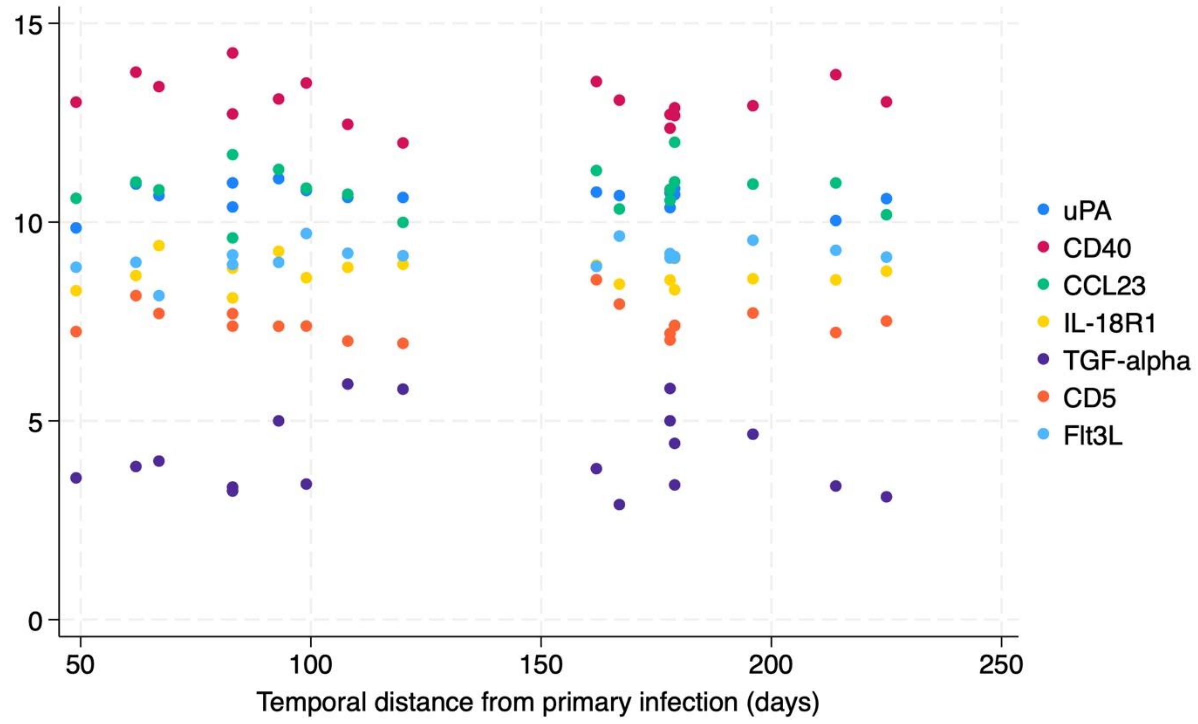

A

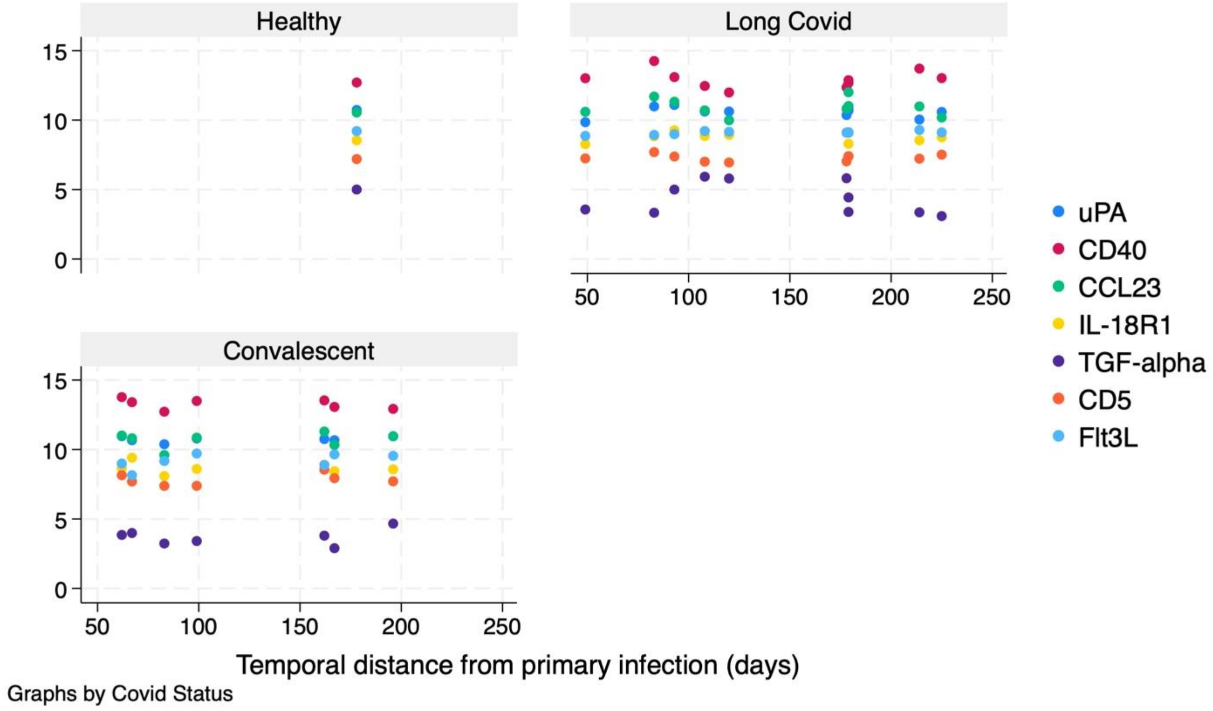

B

Supplemental Digital Content 6. Cytokine profile by persisting symptoms

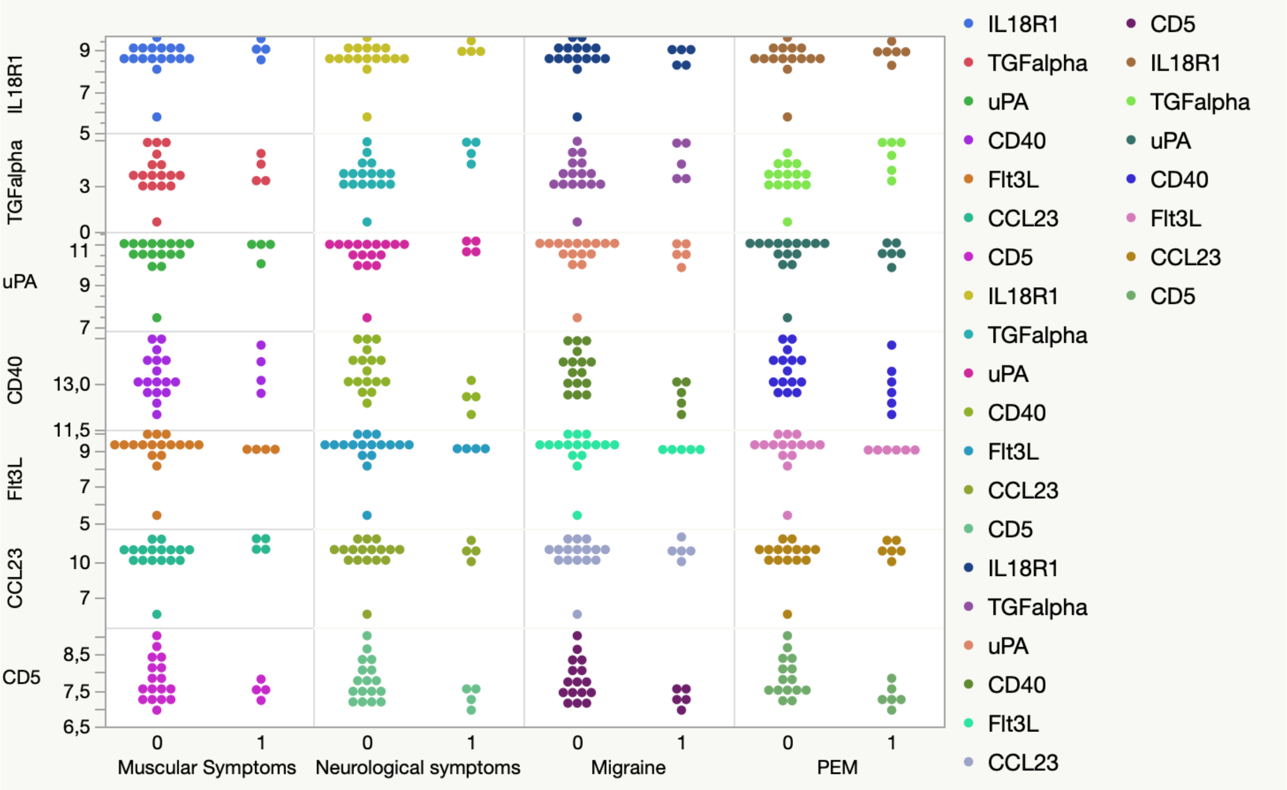

Supplemental Digital Content 7

IL18R1 is the surface marker of CD8+ T cells, being therefore a cytotoxic marker. It resulted higher in patients with Long Covid, suggesting ongoing inflammatory events, in line with our previous study showing imbalance T cell profile in Long Covid (1), but also with other studies in adults (1). Of note, its expression increases with age, a relevant point as our patients were older than controls (2), therefore reinforcing the hypothesis that inflammation can persist after initial SARS-CoV-2 infection. TGFα can also mediate paracrine signaling by activated macrophages, monocytes, neutrophils, and eosinophils, therefore being implicated in inflammatory processes (3).

Flt3 ligand has potent effects on hematopoietic stem cells and the development of the immune system (4). Although its higher expression in Long Covid and convalescent patients can be age related (1), the skewed expression in Long Covid along with its immune effects (the factor is also necessary for NK cells development (5, 6) is of interest and warrants investigations in larger samples of patients.

We found a higher expression of uPA in children after SARS-CoV-2 infection. The uPA system plays an important role in activating the plasmin from its inactive plasminogen form by proteolytic

cleavage (7). Once activated, the plasmin system causes degradation of fibrin, several blood clotting factors, and extracellular matrix (8). In addition, the components of the uPA–uPAR system are involved in the proteolytic activation of several growth factors and cytokines (e.g., basic fibroblast growth factor, bFGF; TGF-β; and IL1β) that are involved in myelopoiesis (9, 10). The uPA system has also been implicated in adaptive immunity. The expressions of both uPA and its receptor uPAR are augmented during T-cell activation compared with their levels in resting or naive T cells (11). In addition, the higher expression of uPA in children after SARS-CoV-2 infections in line with previous observations about the role of uPA as possible biomarker of COVID-19 infection, where lower circulating uPA and uPA/PAI-1 complex levels were more frequent in severely ill patients (12). Moreover, healthy donors show levels of uPA in line for what is expected for their age; on the contrary patients, despite older than healthy donors, had higher circulating uPA (13), reinforcing the correlation of this molecule with the disease.

The lower expression of CD40 is also of interest. CD40 pathways are critical for the generation of an acquired immune response and its deregulation has been observed in multiple autoimmune diseases (14), an interesting finding considering that Long Covid itself has been implicated, along with ME/CFS, among conditions where dysregulated immune responses can play a critical role.
Children with Long Covid showed low levels of CD5, an important physiological regulator of T-cell immune responses (15). The regulation of CD5 corresponds to a key event in the maintenance of immune homeostasis and tolerance. Studies based on experimental mouse models indicates that CD5 plays a key role in generation and maintenance of immune tolerance and that alterations of its activity can promote autoreactivity (16), being in fact the factor associated with autoimmune diseases (17, 18), including children with Systemic Lupus Erythematosus (19). These findings, if confirmed on larger studies, may further reinforce an imbalanced regulation of T cell responses in patients with Long Covid (20).Interestingly, as CD5 and CD40 are widely expressed in healthy donors in line with their age (20, 21), it is interesting to note that patients recovered from both SARS-CoV-2 show a recover of the levels of these molecules, both involved in immune cell proliferation and balance.

References

1- Whiting CC, Siebert J, Newman AM, Du HW, Alizadeh AA, Goronzy J, Weyand CM, Krishnan E, Fathman CG, Maecker HT. Large-Scale and Comprehensive Immune Profiling and

Functional Analysis of Normal Human Aging. PLoS One. 2015 Jul 21;10(7):e0133627. doi: 10.1371/journal.pone.0133627. PMID: 26197454; PMCID: PMC4509650.

2- Zhang L, Hu XZ, Li X, Chen Z, Benedek DM, Fullerton CS, Wynn G; Biomarker team; Ursano RJ. Potential chemokine biomarkers associated with PTSD onset, risk and resilience as well as stress responses in US military service members. Transl Psychiatry. 2020 Jan 23;10(1):31. doi: 10.1038/s41398-020-0693-1. PMID: 32066664; PMCID: PMC7026448.

3- https://www.sciencedirect.com/topics/pharmacology-toxicology-and-pharmaceutical-science/transforming-growth-factor-alpha; accessed on May 1st, 2024

4- Wodnar-Filipowicz A. Flt3 ligand: role in control of hematopoietic and immune functions of the bone marrow. News Physiol Sci. 2003 Dec;18:247-51. doi: 10.1152/nips.01452.2003. PMID: 14614158.

5- Baerenwaldt A., von Burg N., Kreuzaler M., Sitte S., Horvath E., Peter A., Voehringer D., Rolink A.G., Finke D. Flt3 Ligand Regulates the Development of Innate Lymphoid Cells in Fetal and Adult Mice. J. Immunol. 2016;196:2561–2571. doi: 10.4049/jimmunol.1501380. [PubMed] [CrossRef] [Google Scholar]

6- Parigi S.M., Czarnewski P., Das S., Steeg C., Brockmann L., Fernandez-Gaitero S., Yman V., Forkel M., Höög C., Mjösberg J., et al. Flt3 ligand expands bona fide innate lymphoid cell precursors in vivo. Sci. Rep. 2018;8:1–12. doi: 10.1038/s41598-017-18283-0.

7- Mahmood N, Mihalcioiu C and Rabbani SA (2018) Multifaceted Role of the Urokinase-Type Plasminogen Activator (uPA) and Its Receptor (uPAR): Diagnostic, Prognostic, and Therapeutic Applications. Front. Oncol. 8:24. doi: 10.3389/fonc.2018.00024

8- Plesner T, Behrendt N, Ploug M. Structure, function and expression on blood and bone marrow cells of the urokinase-type plasminogen activator receptor, uPAR. Stem Cells (1997) 15(6):398–408. doi:10.1002/stem.150398

9- Naldini L, Tamagnone L, Vigna E, Sachs M, Hartmann G, Birchmeier W, et al. Extracellular proteolytic cleavage by urokinase is required for activation of hepatocyte growth factor/scatter factor. EMBO J (1992) 11(13):4825.

10- Hannocks M-J, Oliver L, Gabrilove JL, Wilson EL. Regulation of proteolytic activity in human bone marrow stromal cells by basic fibroblast growth factor, interleukin-1, and transforming growth factor beta. Blood (1992) 79(5):1178–84.

11- Nykjaer A, Møller B, Todd R, Christensen T, Andreasen PA, Gliemann J, et al. Urokinase receptor. An activation antigen in human T lymphocytes. J Immunol (1994) 152(2):505–16

12- Yatsenko T, Rios R, Nogueira T, Takahashi S, Tabe Y, Naito T, Takahashi K, Hattori K, Heissig B. Urokinase-type plasminogen activator and plasminogen activator inhibitor-1 complex as a serum biomarker for COVID-19. Front Immunol. 2024 Jan 11;14:1299792. doi: 10.3389/fimmu.2023.1299792. Erratum in: Front Immunol. 2024 Mar 13;15:1390698. PMID: 38313435; PMCID: PMC10835145.
13- Weidemann DK, Abraham AG, Roem JL, Furth SL, Warady BA. Plasma Soluble Urokinase Plasminogen Activator Receptor (suPAR) and CKD Progression in Children. Am J Kidney Dis. 2020 Aug;76(2):194-202. doi: 10.1053/j.ajkd.2019.11.004. Epub 2020 Jan 24. PMID: 31987488; PMCID: PMC7374047.
14- Elgueta R, Benson MJ, de Vries VC, Wasiuk A, Guo Y, Noelle RJ. Molecular mechanism and function of CD40/CD40L engagement in the immune system. Immunol Rev. 2009 May;229(1):152-72. doi: 10.1111/j.1600-065X.2009.00782.x. PMID: 19426221; PMCID: PMC3826168.
15- Tabbekh M, Mokrani-Hammani M, Bismuth G, Mami-Chouaib F. T-cell modulatory properties of CD5 and its role in antitumor immune responses. Oncoimmunology. 2013 Jan 1;2(1):e22841. doi: 10.4161/onci.22841. PMID: 23483035; PMCID: PMC3583937.
16- Raman C. CD5, an important regulator of lymphocyte selection and immune tolerance. Immunol Res 2002; 26:255-63; PMID:12403363; http://dx.doi. org/10.1385/IR:26:1-3:255.
17- Berland R, Wortis HH. Origins and functions of B-1 cells with notes on the role of CD5. Annu Rev Immunol. 2002; 20:253–300.
18- Dalloul A. CD5: a safeguard against autoimmunity and a shield for cancer cells. Autoimmun Rev. 2009; 8(4):349–53. [PubMed: 19041428]
19- Asmiyou A, Bakr AM, Shahin DA, Wahba Y. CD40 and CD72 expression and prognostic values among children with systemic lupus erythematosus: a case-control study. Lupus. 2020 Sep;29(10):1270-1276. doi: 10.1177/0961203320941931. Epub 2020 Jul 22. PMID: 32700598.
20- Cevirgel A, Vos M, Holtrop AF, Beckers L, Reukers DFM, Meijer A, Rots N, van Beek J, van Baarle D, de Wit J. Delineating immune variation between adult and children COVID-19 cases and associations with disease severity. Sci Rep. 2024 Mar 1;14(1):5090. doi: 10.1038/s41598-024-55148-9. PMID: 38429462; PMCID: PMC10907598.

21- Kowalczyk D, Macura-Biegun A, Zembala M. The expression of CD40 on monocytes of children with primary humoral immunodeficiencies. Pediatr Res. 2006 Jun;59(6):816-9. doi: 10.1203/01.pdr.0000219298.96471.18. Epub 2006 Apr 26. PMID: 16641210.
